# Outcomes of five cases of retinoblastoma with optic nerve invasion on imaging

**DOI:** 10.1007/s10384-024-01112-z

**Published:** 2024-09-28

**Authors:** Tamae Onishi, Sachiko Nishina, Tadashi Yokoi, Tomoyo Yoshida, Shion Hayashi, Hazuki Morikawa-Anzai, Noriyuki Azuma, Chikako Kiyotani, Keita Terashima, Takako Yoshioka, Hideki Ogiwara, Hiroshi Fuji, Masayuki Kitamura, Yoshiyuki Tsutsumi

**Affiliations:** 1 Division of Ophthalmology, National Center for Child Health and Development, 2-10-1 Okura Setagaya-ku, Tokyo, Japan; 2 Children’s Cancer Center, National Center for Child Health and Development, Tokyo, Japan; 3 Department of Pathology, National Center for Child Health and Development, Tokyo, Japan; 4 Division of Neurosurgery, National Center for Child Health and Development, Tokyo, Japan; 5 Division of Radiation Oncology, National Center for Child Health and Development, Tokyo, Japan; 6 Department of Diagnostic Radiology, National Center for Child Health and Development, Tokyo, Japan

**Keywords:** Retinoblastoma, Optic nerve invasion, Enucleation, Magnetic resonance imaging, Neoadjuvant chemotherapy

## Abstract

**Purpose:**

To investigate the timing of enucleation, treatment course, and outcome for retinoblastoma (RB) with optic nerve (ON) invasion on imaging.

**Study design:**

Retrospective clinical study.

**Methods:**

Of the 160 patients with RB who presented to the National Center for Child Health and Development in Japan between 2005 and 2022, ON invasion on imaging at the initial presentation was seen in five patients. The clinical, computed tomography (CT), and magnetic resonance imaging (MRI) findings, and treatment courses were reviewed retrospectively.

**Results:**

MRI showed ON invasion in all five patients (three with unilateral RB, 2 with bilateral RB); in two patients CT detected no invasion. Enucleation was performed in four patients, three of whom underwent neoadjuvant therapy and one had a positive ON resection margin following the enucleation as initial treatment. One patient did not undergo enucleation due to cerebrospinal fluid dissemination. All enucleated patients underwent adjuvant chemotherapy. Four patients underwent radiotherapy. During follow-up (mean, 89.4 months), four patients survived and one died.

**Conclusion:**

MRI is recommended to evaluate ON invasion and determine the timing of enucleation for RB. The appropriate choice of neoadjuvant or adjuvant therapy would be helpful to avoid radiotherapy for RB with ON invasion on imaging.

## Introduction

Retinoblastoma (RB) is the most common ocular malignant tumor in children [1]. Extraocular extension, especially optic nerve (ON) invasion is the worst factor affecting survival [2]. Although due to alternative therapy for less advanced RB enucleation is now performed less frequently [3], it remains an important option for advanced RB. The additions of neoadjuvant chemotherapy and radiotherapy to the standard treatment for RB with extraocular invasion is not determined [4]. The survival rates for RB with extraocular invasion ranges from 40 to 80% [5–9]. We investigated the timing of enucleation and course of treatment for RB with ON invasion in five patients.

## Materials and methods

The ethics committee of the National Center for Child Health and Development (NCCHD) approved this study (permit no. 2022-017). The legal guardians of the five patients and one patient over 18 years old provided informed consent. The medical records of patients diagnosed at the NCCHD between 2005 and 2022 for RB with ON invasion were reviewed retrospectively. The inclusion criteria were the presence of ON invasion based on computed tomography (CT) and/or magnetic resonance imaging (MRI). The data collected included age, sex, B-mode ultrasonography, CT and MRI findings, pathological findings, and treatment courses. Between 2005 and 2022, 160 patients with RB were diagnosed at the NCCHD. Five patients (three boys, two girls) met the inclusion criteria. The mean age at diagnosis was 15.8 months (range, 3–22) and the mean follow-up was 89.4 months (range, 11–209).

## Results

### Clinical ophthalmologic and radiologic features

**Fig. 1:**
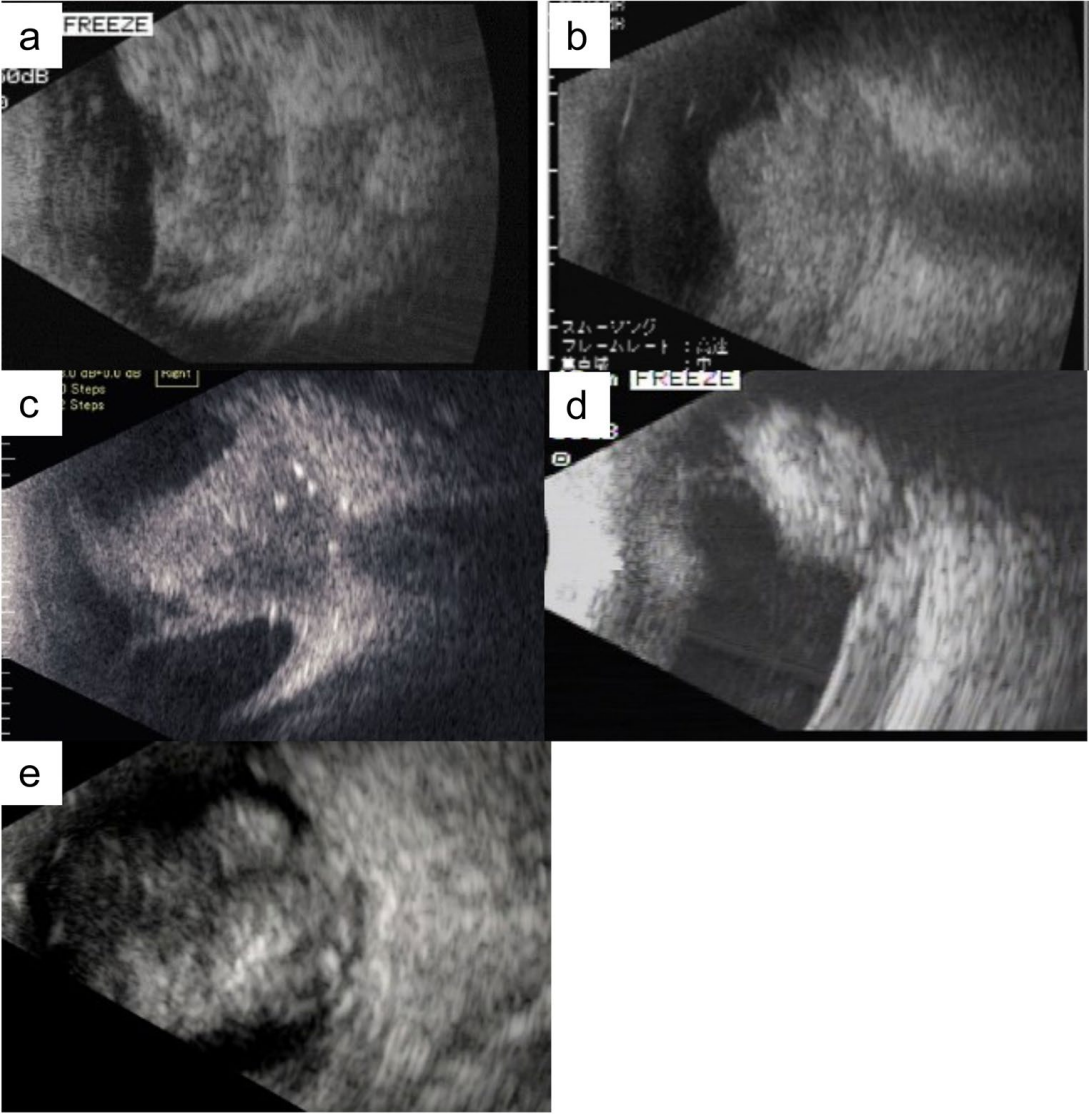
B-mode ultrasonograms of cases 1–5 (**a**-**e**). A solid tumor mass with highly reflective foci is seen in each case, but no obvious invasion of the optic nerve (ON) is detected in any case

**Fig. 2:**
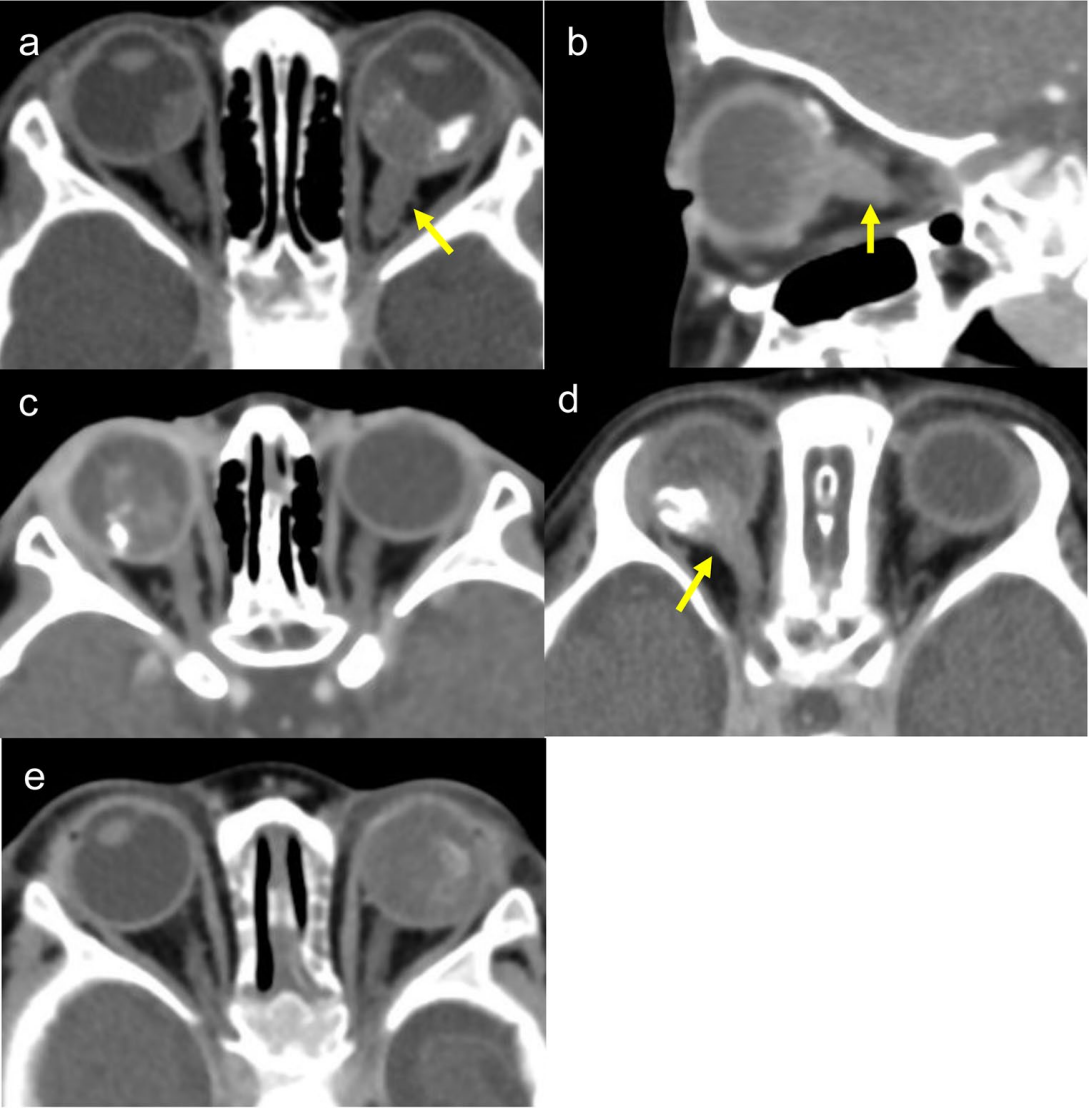
Contrast-enhanced computed tomography (CT) images of Cases 1–5 (**a**-**e**). ON swelling and contrast enhancement indicating ON invasion (arrows) are seen. There is no evidence of ON invasion (**c**, **e**)

**Fig. 3:**
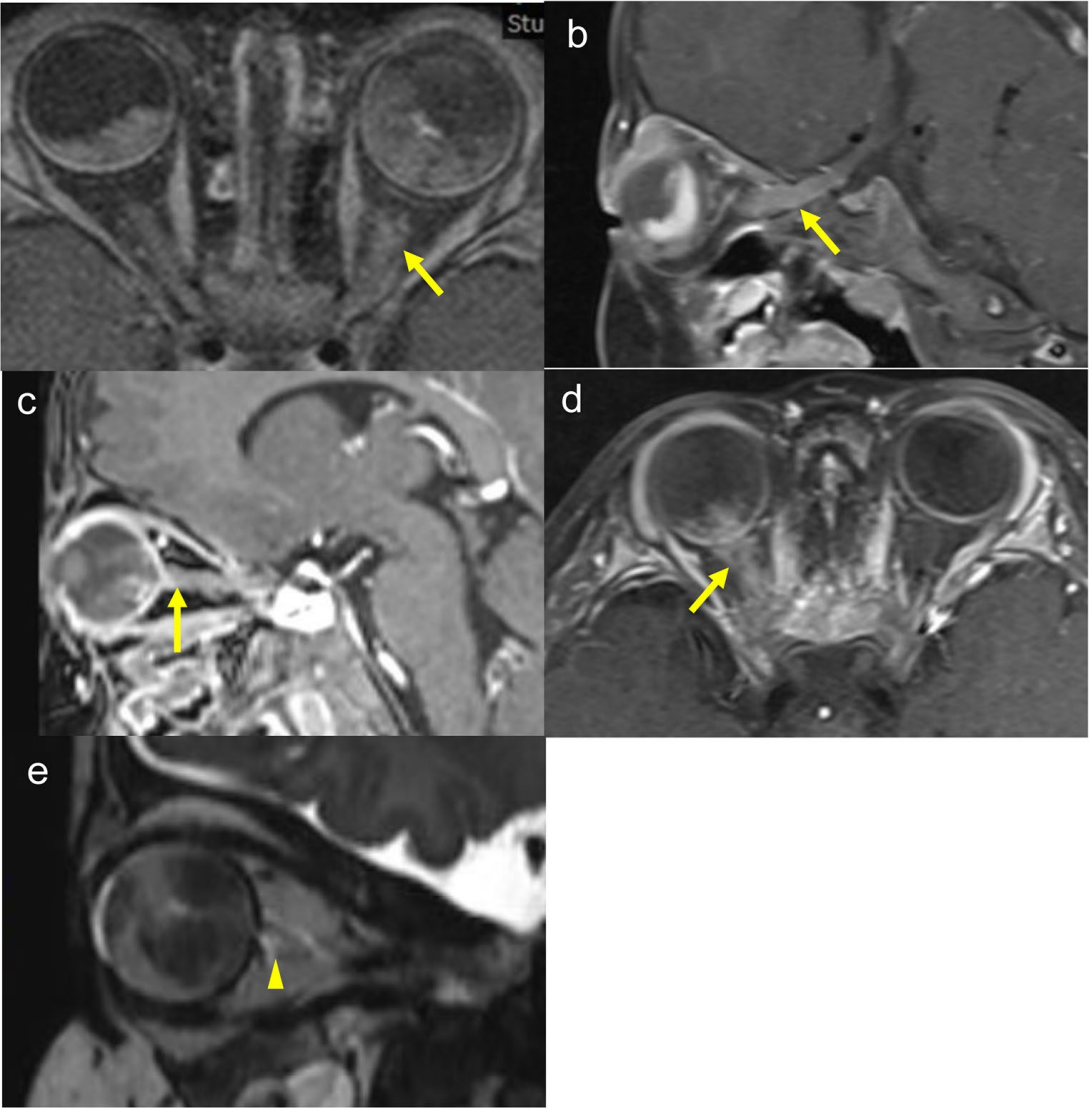
Contrast magnetic resonance imaging (MRI) images of case 1–4 (**a**-**d**) and MRI SPACE image of case 5 (**e**). ON contrast enhancement shows ON invasion (arrows). An enlarged origin of the left ON with disruption of the surrounding subarachnoid space indicates ON invasion (arrowhead)

Three patients presented with the chief complaint of leukocoria and two patients with eyelid swelling. Two patients had bilateral RB and three patients had unilateral RB. B-mode ultrasonography of all patients showed solid tumor masses, but there was no obvious invasion of the ON (Fig. 1). All five patients underwent whole-body CT and head MRI imaging as the initial radiologic investigation at the NCCHD before treatment. CT did not detect ON invasion in two patients, whereas MRI showed ON invasion in all patients (Figs. 2 and 3). Cerebrospinal fluid (CSF) cytology was performed for all patients before or during treatment. Bone marrow biopsy was done for four patients (cases 2–5) before treatment. Case 2 showed CSF positivity before treatment, and case 4 showed CSF positivity after enucleation. The bone marrow biopsies were all negative.

The pre-treatment findings in the five cases are summarized in Table 1. The specific clinical courses and radiologic features of the five cases are provided below.

**Table 1:** Pre-treatment findings

Case	1	2	3	4	5
Age at diagnosis (months)	18	15	21	22	3
Affected side	R/L	L	R	R	R/L
ICRB group (R/L)	D/E	E	E	E	A/E
Eyelid swelling (R/L)	−/−	−/+	+/−	−/−	−/−
ON invasion (CT) (R/L)	−/+	−/+	−/−	+/−	−/−
ON invasion (MRI) (R/L)	−/+	−/+	+/−	+/−	−/+
Length and place of invasion on MRI	13 mm, in front of optic canal	31 mm, in front of optic chiasm	3 mm, anterior end of ON	19 mm, in front of optic canal	5 mm, anterior end of ON
Metastasis (bone marrow)	NA	−	−	−	−
Metastasis (CSF)	−	+	−	−	−
TNM classification	T4aN0M0	T4aN0M1b	T4aN0M0	T4aN0M0	T4aN0M0

*R* right eye; *L* left eye; *TNM* tumor-node-metastasis; *ON* optic nerve; *ICRB* International Classification for Retinoblastoma; *NA* not applicable

### Case 1

An 18-month-old female infant with leukocoria OS was referred to our center with suspected bilateral RB. At the initial examination, anisocoria was observed and the left eye showed mydriasis and leukocoria. Ophthalmoscopy showed that the RB extended from the inferior to nasal retina, but the macula was not involved in the right eye. In the left eye, the RB extended throughout the fundus. B-mode ultrasonography showed a large solid mass with highly reflective foci OS (Fig. 1a) and a small mass OD. There was no obvious ON invasion on ultrasonography. The right eye was classified in Group D and the left eye in Group E of the International Classification of Retinoblastoma (ICRB) [10]. CSF cytology was negative before treatment. A bone marrow biopsy was not performed in this case.

Brain CT showed an intraocular tumor with bilateral calcification. The left ON was thicker than the right and showed contrast enhancement (Fig. 2a). On MRI, the left ON showed contrast enhancement and swelling, and the RB infiltrated the ON by 13 mm, which is in front of the optic canal on imaging. There was no obvious invasion in the right ON (Fig. 3a).

### Case 2

A 15-month-old male infant with swelling of the left upper eyelid was referred to the NCCHD with suspected RB OS. At the initial examination, the left eye showed leukocoria. The fundus of the left eye was not visible. The right eye was normal. B-mode ultrasonography showed a large solid mass OS (Fig. 1b). The ON invasion was not obvious on ultrasonography.

CT showed an intraocular tumor OS. The left ON was obviously thicker than the right and showed contrast enhancement that continued intracranially (Fig. 2b). MRI showed the contrast effect in the left ON with 31 mm of infiltration to just in front of the intracranial optic chiasm (Fig. 3b). A biopsy performed to rule out an ON glioma and hamartoma identified RB. We performed lensectomy and vitrectomy using the limbal approach. Specimens were obtained from the tumor mass. Considering the possibility of surgical dissemination, the patient underwent transcorneal surgery without vascular structures. The left eye was classified in Group E of the ICRB. CSF cytology was positive before treatment and a bone marrow biopsy was negative.

### Case 3

A 21-month-old female infant with eyelid swelling of her right upper eyelid was referred to our center. The anterior segment OD showed inflammation, iris rubeosis, and synechia. During the fundus examination, the right eye could not be evaluated because of vitreous opacity and the left eye was normal. B-mode ultrasonography showed a solid mass with highly reflective foci OD (Fig. 1c). No ON invasion was obvious on B-mode ultrasonography. The right eye was classified in Group E of the ICRB. CSF cytology and bone marrow biopsy were negative before treatment.

On CT, an intraocular tumor with contrast enhancement and calcification was seen OD. The right ON has no contrast enhancement or swelling (Fig. 2c). MRI showed contrast enhancement of the right ON and posterior wall of the right eye, with infiltration to 3 mm of the anterior end of the ON (Fig. 3c).

### Case 4

A 22-month-old male infant with leukocoria OD was referred to our center. The examination showed corneal edema and iris rubeosis OD. The fundus of the right eye could not be visualized. The fundus of the left eye was normal. B-mode ultrasonography showed a mass with a highly reflective area OD (Fig. 1d). No ON invasion was obvious on B-mode ultrasonography. The right eye was classified in Group E of the ICRB. CSF cytology was negative initially and became positive after enucleation. A bone marrow biopsy was negative.

CT showed an intraocular tumor with contrast enhancement and calcification OD. The right ON was thicker than the left and showed a contrast effect (Fig. 2d). MRI showed contrast enhancement of the right ON with infiltration of 19 mm in front of the optic canal in the orbit (Fig. 3d).

### Case 5

A 3-month-old male infant with leukocoria OS was referred to our center. At the examination, the tumor touched the posterior lens capsule. Fundus examination showed RBs on both fundi. Small tumors were scattered through the right fundus, but the macula was intact. The left fundus was covered by tumor. Fluorescein angiography showed five small tumors on the right fundus. B-mode ultrasonography showed a large mass with a highly reflective area OS (Fig. 1e), but it could not detect a tumor mass OD. The right eye was classified in Group A and the left eye in Group E of the ICRB. CSF cytology and bone marrow biopsy were negative before treatment.

CT showed an intraocular tumor with calcification OS, but no obvious tumor was detected OD. A contrast effect and swelling were not detected in either ON (Fig. 2e). An MRI sampling perfection with application-optimized contrasts using different flip angle evolutions (SPACE) image showed that the origin of the left ON was enlarged with disruption of the surrounding subarachnoid space. RB infiltration of 5 mm into the anterior end of the left ON was observed (Fig. 3e).

### Primary treatment

Basically, we try to perform neoadjuvant chemotherapy in cases with ON invasion and then perform secondary enucleation when MRI no longer shows ON invasion. However, case 1 had presented before this strategy was established and was preceded by enucleation. Four patients (cases 2–5) underwent chemotherapy as the primary treatment. Three of four patients (cases 3–5), who primarily underwent chemotherapy ultimately underwent enucleation, while one patient (case 2) could not undergo enucleation due to consistent ON invasion seen on MRI, positive results of CSF, and progression of liver metastasis. Case 5 with bilateral RB underwent photocoagulation for small tumors of the right fundus; enucleation was not planned for this eye.

The chemotherapy regimens differed in each case. At NCCHD, the regimens for RB with ON invasion are basically the same as those for medulloblastoma [11–13]. Intrathecal injection (IT) also was basically used according to these regimens, but in some cases, IT was performed at the same time as the CSF examination to determine the efficacy of treatment for positive CSF status. The treatments of the five cases are summarized in Tables 2 and 3.

**Table 2:** Treatment of 5 cases

Case	1	2	3	4	5
Primary treatment	Enucleation	Chemotherapy	Chemotherapy	Chemotherapy	Chemotherapy
Timing of enucleation	1 week after diagnosis	NA	After 2 courses of chemotherapy	After 4 courses of chemotherapy	After 3 courses of chemotherapy
Method of enucleation	Anterior approach	NA	Anterior approach	Anterior approach	Double approach
Surgical margin	Positive	NA	Negative	Negative	Negative
Pathological findings	Tumor cells in epidural tissue	Small cluster formed by small round cells	Tumor cells in ON and choroid	No viable tumor cells in ON, CSF (+)	No viable tumor cells in ON
Transplant	Auto-BMT	Auto-PBSCT	No	auto PBSCT	No
Radiotherapy	EBRT Right eye Left orbit 40 Gy	EBRT Brain Spinal cord Liver Left eye 45 Gy	EBRT Right orbit Optic chiasm 45 Gy	EBRT Brain Spinal cord Right orbit 45 Gy	No
Follow-up (months)	209	11 (death)	116	98	13

*Auto-BMT* autologous bone marrow transplant; *ON* optic nerve; *auto-PBSCT* autologous peripheral blood stem cell transplantation; *EBRT* external beam radiotherapy; *CSF* cerebrospinal fluid; *NA* not applicable

**Table 3:** Chemotherapy

Case	1	2	3	4	5
Neoadjuvant chemotherapy	NA	ICE + VCR + IT^*5^ VCR + IT^*6^ VCR + CY + CDDP + ETP + IT^*7^ VEC + IT^*8^ 2×VCR^*9^ VCR + CY + CDDP + ETP^*10^ 2×IT^*11^ VEC^*12^ IT^*11^ IT^*13^ 3×Topo + CBDCA^*14^	2×JPBTC MB/PNET regimen^*15^	4×VEC^*16^ 3×IT^*17^	3×VEC^*16^ VCR^*9^ IT^*22^ 2×IT^*23^
Adjuvant chemotherapy regimen	VCR + CY + CDDP + ETP + IT^*1^ VCR + CY + CDDP^*2^ VCR^*3^ VCR + CY + CDDP + ETP + IT^*1^ VCR + CY + CDDP^*2^	NA	4×VEC^*16^	VEC^*16^ 6×IT^*18^ Packer regimen ^*19^ 4×JPBTC MB/PNET regimen^*20^	3× ICE^*24^ 3×IT^*23^
High-dose chemotherapy	TEPA + MEL^*4^	No	No	TEPA + MEL^*21^	No

*1. VCR 1.5 mg/m^2^ D1 + CY 1 g/m^2^ D1, 3, 5 + CDDP 90 mg/m^2^ D2 + ETP 100 mg/m^2^ D1-5 + IT (MTX 8 mg + DEX 5 mg) [11]

*2. VCR 1.5 mg/m^2^ D1 + CY 1 g/m^2^ D1, 3, 5 + CDDP 90 mg/m^2^D2 [11]

*3. VCR 1.5 mg/m^2^

*4. TEPA 8 mg/kg D1, 2, 8 ,9 + MEL 1.5 mg/m^2^ D1, 2, 8, 9

*5. IFO 1.8 mg/m^2^ D2-5 + CBDCA 18.6 mg/kg D1 + ETP 5 mg/kg D1-5 + VCR 0.05 mg/kg D1,8 + IT (MTX 8 mg + DEX 5 mg) D1,8

*6. VCR 1.5 mg/m^2^ + IT-MD (MTX 8 mg + DEX 5 mg)

*7. VCR 0.05 mg/kg D1,8 + CY 33 mg/kg D1, 3, 5 + CDDP 3 mg/kg D2 + ETP 3.3 mg/kg D1-5 + IT (MTX 8 mg + DEX 5 mg) D1, 8 [11]

*8. VCR 0.05 mg/kg D1 + ETP 3.3 mg/kg D1,2 + CDDP 3 mg/kg D1 + IT-MD (MTX 8 mg + DEX 5 mg)

*9. VCR 0.05 mg/kg

*10. VCR 0.05 mg/kg D1 + CY 33 mg/kg D1, 3, 5 + CDDP 3 mg/kg D2 + ETP 3.3 mg/kg D1-5

*11. IT (Ara-C1 5 mg + MTX 7.5 mg + HDC 15 mg)

*12. VCR 1.5 mg/m^2^ D1 + ETP 150 mg/m^2^ D1, 2 + CDDP 560 mg/m^2^ D1

*13. IT (MTX 8 mg + DEX 5 mg)

*14. Topo 2 mg/30 kg D1-5 + CBDCA 400 mg/30 kg D7-8

*15. VCR 0.05 mg/kg D1, 8, 15 + CY 33 mg/kg D1, 3, 5 + CDDP3 mg/kg D2 + ETP 3.3 mg/kg D1-5 [11]

*16. VCR 0.05 mg/kg D1 + ETP 5 mg/kg D1,2 + CBDCA 18.6 mg/kg D1

*17. IT (MTX 8 mg + PSL 6 mg)

*18. IT (Topo 0.32 mg + Ara-C 24 mg)

*19. VCR 1.5 mg/m^2^ D1, 8, 15 + CY 1 g/m2 D22,23 + CDDP 75 mg/m^2^ D1 [12]

*20. VCR 1.5 mg/m^2^ D1 + CY 1 g/m^2^ D1,3,5 + CDDP 90 mg/m^2^ D2 [11]

*21. TEPA800 mg/m^2^ D1, 2, 8, 9 + MEL 210 mg/m^2^ D1, 2, 8, 9

*22. IT (MTX 6 mg + HDC 15 mg)

*23. IT (MTX 15 mg/m^2^ + AraC 60 mg/m^2^ + HDC 30 mg/m^2^)

*24. IFO 60 mg/kg D1-5 + CBDCA 13.3 mg/kg D1,2 + ETP 3.3 mg/kg D1-5 [13]

*VCR* vincristine; *CY* cyclophosphamide; *CDDP* cisplatin; *ETP* etoposide; *IT* intrathecal injection; *MTX* methotrexate; DEX dexamethasone; *IFO* ifosfamide; *CBDCA* carboplatin; *HDC* hydrocortisone; *Topo* topotecan; *Ara-C* Cytarabine; *VEC* vincristine, etoposide, carboplatin; *JPBTC MB/PNET* regimen Japanese Pediatric Brain Tumor Consortium medulloblastoma/primitive neuroectodermal tumors regimen; vincristine, cisplatin, cyclophosphamide; *ICE* ifosfamide, carboplatin, etoposide; *TEPA* thiotepa; *MEL* melphalan; *auto BMT* autologous bone marrow transplant; *auto PBSCT* autologous peripheral blood stem cell transplantation; *EBRT* external beam radiotherapy; *NA* not applicable

### Surgical treatment

Of the patients who underwent enucleation, the ON surgical margin was positive in case 1, which preceded enucleation. In cases 3–5, we checked for the absence of ON invasion on MRI after neoadjuvant chemotherapy and then performed enucleation. The ON surgical margins were negative in all of them. Case 5 underwent enucleation using a double approach [8] that included both the anterior and neurosurgical approaches to ensure that ON surgical margins were negative.

### Histopathologic results

**Fig. 4:**
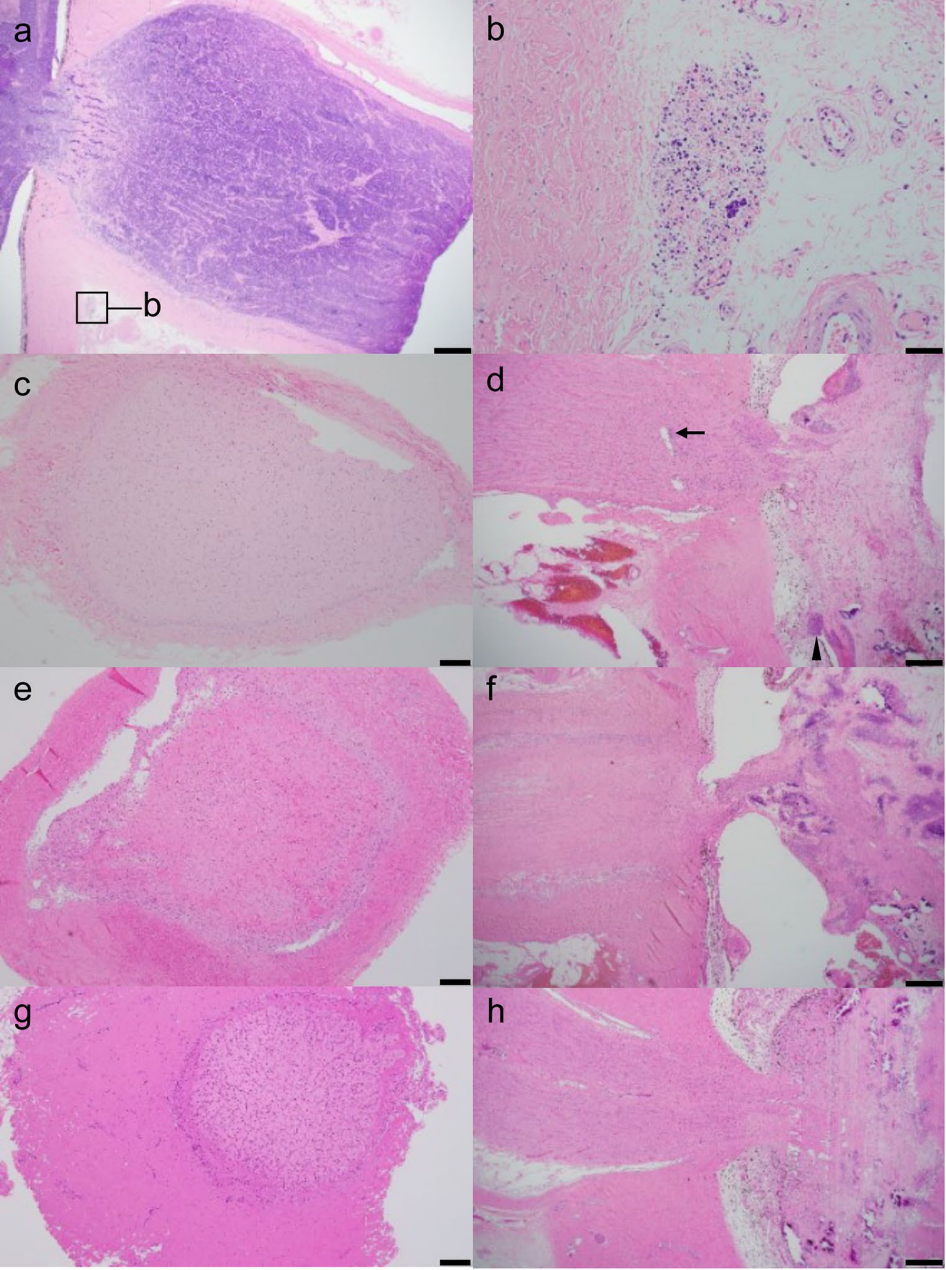
Histopathological images of case 1 (**a**, **b**), case3 (**c**, **d**), case 4 (**e**, **f**) and case 5 (**g**, **h**). a ON with tumor invasion. Tumor cells observed in epidural tissue (box). Hematoxylin and Eosin (H&E) staining. Bar = 500 μm. b Tumor cells in epidural tissue. H&E staining. Bar = 50 μm. **c** No viable tumor cells are seen in the surgical margin. H&E staining. Bar = 200 μm. **d** ON showed fibrotic changes. Tumor cells found in the ON (arrow) and the choroid (arrowhead). H&E staining. Bar = 500 μm. **e** No viable tumor cells are seen in the surgical margin. H&E staining. Bar = 200 μm. **f** ON with fibrotic change. H&E staining. Bar = 500 μm. **g** ON with negative surgical margin. H&E staining. Bar = 200 μm. **h** ON retracted into the eye. Arachnoid space and ON without viable cells. H&E staining. Bar = 500 μm

All enucleated eyes were examined pathologically. In case 1, the tumor cells were continuously infiltrating beyond the cribriform plate to the surgical margin (Fig. 4a). Tumor cells were observed in the epidural tissue (Fig. 4b). In case 2, the biopsy established a diagnosis of RB, and small round cells formed a small cluster. In case 3, the ON showed fibrotic changes due to neoadjuvant chemotherapy. No viable tumor cells were seen in the surgical margin (Fig. 4c) or arachnoid space, while there were tumor cells in the ON and rosettes within the choroid (Fig. 4d). In case 4, the ON showed fibrotic changes due to neoadjuvant chemotherapy. No viable tumor cells were seen in the surgical margin (Fig. 4e), ON, or arachnoid space (Fig. 4f). In case 5, no viable tumor cells were seen in the ON, surgical margin (Fig. 4g), or arachnoid space. The ON was retracted into the eye due to neoadjuvant chemotherapy (Fig. 4h).

### Adjuvant treatment

All enucleated patients underwent chemotherapy postoperatively based on a high-risk preoperative evaluation that there was ON invasion. High-dose chemotherapy was administered to the cases with a positive ON margin or positive CSF. Case 1 had a positive ON surgical margin, and case 4 had a positive CSF after enucleation. When high-dose chemotherapy was administered, autologous bone marrow transplantation or autologous peripheral blood stem cell transplantation was performed.

### Radiotherapy

Cases 1-4 were treated with radiotherapy (total dose of external beam radiotherapy, 40–45 Gy). Radiotherapy was administered to the orbit whenever the disease was contained within the orbit and to the whole brain and spinal cord if dissemination to the CSF had occurred. Radiotherapy was administered to distant metastases (the liver in case 2).

Case 5 with bilateral RB did not undergo radiotherapy; MRI was performed and showed 5 mm of ON invasion and the patient achieved a negative surgical margin and no residual viable tumor cells in the pathological findings. The patient was younger than 1 year and had bilateral RB that seemed to have a germline mutation. Therefore, case 5 was at high risk for a secondary cancer.

### Treatment-related toxicity

Late complications included central endocrine abnormalities and cataracts due to radiotherapy in cases 1 and 4. Neurologic symptoms due to multiple cerebral infarctions and hemorrhage developed in case 2. No relapse or second cancer occurred in any cases.

### Prognoses

Four patients were alive at the last follow-up. Case 2, which had intracranial invasion seen at the initial diagnosis, died 11 months after the initial diagnosis (Table 2).

## Discussion

Although numerous reports have been published regarding the poor prognoses of RB with ON invasion, the treatment has not been standardized. We summarized the previously published reports of RB with extraocular invasion (Table 4). The rate of radiotherapy and survival rates vary among the reports. Depending on the report, there are cases with and without extraocular invasion on imaging evaluation. All reports that preceded enucleation in Table 4, except for that of Choucair et al. [8] and Bellaton et al. [14], describe no extraocular invasion on the preoperative imaging evaluation, but postoperative pathological evaluation showed extraocular invasion and adjuvant chemotherapy was administered. However, if preoperative imaging showed extraocular invasion, neoadjuvant chemotherapy was either administered or excluded in these reports. Choucair et al. report a relatively low rate of radiotherapy (45.5%) and a good survival rate (70%) for patients with ON invasion on imaging, similar to the current subjects; their patients were treated with neoadjuvant chemotherapy and the double approach enucleation [8].

**Table 4:** Summary of reports

Reports	Stage	No. cases	No. of extraocular invasions on CT or MRI	Treatment and no. of cases		Radiation therapy	Survival rate (follow-up period)
Chantada et al. [5]	IRSS Stages II, III	26	5 (CT or MRI)	Enucleation + adjuvant chemotherapy	21	26 cases (100%)	70% (5 years)
				Neoadjuvant chemotherapy + enucleation + adjuvant chemotherapy	5		
Radhakrishnan et al. [6]	IRSS Stage III	28	28 (MRI)	Neoadjuvant chemotherapy + enucleation + adjuvant chemotherapy	21	19 cases (67.9%)	40.4% (14.75 months)
				Neoadjuvant chemotherapy + enucleation	1		
				Chemotherapy	6		
Künkele et al. [7]	IRSS Stage II	6	1 (MRI)	Enucleation + adjuvant chemotherapy	5	6 cases (100%)	80% (5 years)
				Neoadjuvant chemotherapy + enucleation + adjuvant chemotherapy	1		
Choucair et al. [8]	ON invasion on CT or MRI	11	11 (MRI)	Enucleation + adjuvant chemotherapy	1	5 cases (45.5%)	70% (2 years)
				Enucleation + adjuvant chemotherapy + high-dose chemotherapy	1		
				Neoadjuvant chemotherapy + double approached enucleation + adjuvant chemotherapy	7		
				Neoadjuvant chemotherapy + enucleation + adjuvant chemotherapy	1		
				Chemotherapy	1		
Zhao et al. [9]	IRSS Stage II	22	0	Enucleation + adjuvant chemotherapy	22	3 cases (13.6%)	40.9% (5 years)
Bellaton et al. [14]	ON invasion on CT or MRI	4	4 (CT or MRI)	Neoadjuvant chemotherapy + double approached enucleation + adjuvant chemotherapy	3	0 case (0%)	75% (12–40 months)
				Chemotherapy	1		

*IRSS* International retinoblastoma staging system

A limitation of the current report is that the five cases were treated at different times (from 2005 to 2022) and with different chemotherapy regimens. Over time, at NCCHD more emphasis has been placed on neoadjuvant chemotherapy for extraocular invasion. Although we used the pathology results after neoadjuvant chemotherapy as a guide, we also performed adjuvant therapy based on a high-risk preoperative evaluation that showed ON invasion. Because we tried to avoid radiation therapy as much as possible and the risk of down-staging, we planned adjuvant therapy for high-risk cases with ON invasion. If the margins are positive, radiotherapy is definitely needed. We prefer to avoid radiotherapy for secondary cancer prevention, especially in young patients with bilateral RB who seemed to have a germline mutation.

Bellaton et al. report neoadjuvant chemotherapy and double approach enucleation in 3 patients with ON invasion seen on imaging, and no patients received radiotherapy [14]. Choucair et al. report that of the 7 patients who underwent neoadjuvant chemotherapy and double approach enucleation, 2 patients underwent radiotherapy. One patient who underwent neoadjuvant chemotherapy and anterior enucleation was positive with a surgical margin of 5 mm and underwent postoperative radiation therapy [8]. No major complications, such as infection, developed in these reports. In addition, the small size of the orbit in young patients occasionally may cause difficulty in removing the ON margin by an anterior approach as in case 5, even though it was 5 mm. Although the double approach was invasive, we avoided radiotherapy, at least in the young patient in case 5 who had bilateral RB and seemed to have a germline mutation. Although the number of current cases is too small to be definitive, it is possible that the double approach may help patients avoid radiation therapy.

We performed secondary enucleation only after confirming the negative results of ON invasion on MRI. Chawla et al. report a high negative predictive rate (100%) in determining ON invasion by MRI after neoadjuvant chemotherapy [15]. Therefore, when MRI after neoadjuvant chemotherapy shows no ON invasion, enucleation is recommended. However, in case 4, the ON invasion progressed in front of the optic canal and the CSF was positive postoperatively. Even though MRI showed no ON invasion after neoadjuvant chemotherapy, the CSF could not be evaluated. In cases with intracranial invasion, the prognosis is poor, and the choice of treatment remains an issue.

B-mode ultrasonography was helpful for detecting the presence of tumor at the primary diagnosis when the fundus cannot be evaluated. However, it could not detect ON invasion in all 5 cases and was inferior to MRI and CT for diagnosing ON invasion.

Considering the differences in results of ON invasion between CT and MRI images from cases 3 and 5, compared to MRI CT may not be able to detect ON invasion adequately. Although the MRI findings were correlated with the pathology results, when compared the positive predictive values of CT is reported to be 66.67% [16]. Gadolinium enhancement on MRI is essential. In the current study, ON invasion also was found by an enhancement effect in all cases. Some studies report that the contrast patterns were correlated with pathology [17] and that MRI after chemotherapy can evaluate ON invasion [15]. In the current study, 133 eyes of 130 patients underwent enucleation after imaging without ON invasion. Of these, no eyes had a positive surgical ON margin, and 13 eyes of 13 cases (9.8%) had laminar cribrosa invasion pathologically. Only 5 of the 13 cases were evaluated by MRI imaging, while the remaining 8 cases were only evaluated by CT imaging.

Considering the current outcomes of the 5 cases, enhanced MRI, rather than CT is recommended to evaluate ON invasion. For RB with ON invasion observed on imaging, appropriate selection of neoadjuvant or adjuvant therapy would be helpful to avoid radiotherapy, especially for young patients or for those with a germline mutation.
